# Functional characterization of human Cd33^+ ^And Cd11b^+ ^myeloid-derived suppressor cell subsets induced from peripheral blood mononuclear cells co-cultured with a diverse set of human tumor cell lines

**DOI:** 10.1186/1479-5876-9-90

**Published:** 2011-06-09

**Authors:** Melissa G Lechner, Carolina Megiel, Sarah M Russell, Brigid Bingham, Nicholas Arger, Tammy Woo, Alan L Epstein

**Affiliations:** 1 Department of Pathology, USC Keck School of Medicine, Los Angeles, California, USA

**Keywords:** myeloid-derived suppressor cells, tumor immune tolerance, human tumor cell lines, immunomodulation, cytokines, hypoxia-inducible factor 1 alpha, CAAAT-enhancer binding protein, signal transducer and activator of transcription, inflammation

## Abstract

**Background:**

Tumor immune tolerance can derive from the recruitment of suppressor cell populations, including myeloid-derived suppressor cells (MDSC). In cancer patients, MDSC accumulation correlates with increased tumor burden, but the mechanisms of MDSC induction remain poorly understood.

**Methods:**

This study examined the ability of human tumor cell lines to induce MDSC from healthy donor PBMC using *in vitro *co-culture methods. These human MDSC were then characterized for morphology, phenotype, gene expression, and function.

**Results:**

Of over 100 tumor cell lines examined, 45 generated canonical CD33^+^HLA-DR^low^Lineage^- ^MDSC, with high frequency of induction by cervical, ovarian, colorectal, renal cell, and head and neck carcinoma cell lines. CD33^+ ^MDSC could be induced by cancer cell lines from all tumor types with the notable exception of those derived from breast cancer (0/9, regardless of hormone and HER2 status). Upon further examination, these and others with infrequent CD33^+ ^MDSC generation were found to induce a second subset characterized as CD11b^+^CD33^low^HLA-DR^low^Lineage^-^. Gene and protein expression, antibody neutralization, and cytokine-induction studies determined that the induction of CD33^+ ^MDSC depended upon over-expression of IL-1β, IL-6, TNFα, VEGF, and GM-CSF, while CD11b^+ ^MDSC induction correlated with over-expression of FLT3L and TGFβ. Morphologically, both CD33^+ ^and CD11b^+ ^MDSC subsets appeared as immature myeloid cells and had significantly up-regulated expression of iNOS, NADPH oxidase, and arginase-1 genes. Furthermore, increased expression of transcription factors HIF1α, STAT3, and C/EBPβ distinguished MDSC from normal counterparts.

**Conclusions:**

These studies demonstrate the universal nature of MDSC induction by human solid tumors and characterize two distinct MDSC subsets: CD33^+^HLA-DR^low^HIF1α^+^/STAT3^+ ^and CD11b^+^HLA-DR^low^C/EBPβ^+^, which should enable the development of novel diagnostic and therapeutic reagents for cancer immunotherapy.

## Background

Myeloid-derived suppressor cells (MDSC) have recently been recognized as a subset of innate immune cells that can alter adaptive immunity and produce immunosuppression [1]. In mice, MDSC are identified by CD11b^+^, IL-4Rα^+^, and GR-1^low/int ^expression, with recognized granulocytic and monocytic subsets [2-6]. Human MDSC are less understood and comprise a heterogeneous population of immature myeloid (CD33^+^) cells consisting of dendritic cell, macrophage, and granulocyte progenitors that lack lineage maturation markers [2,5]. MDSC inhibit T cell effector functions through a range of mechanisms, including: arginase 1 (ARG-1)-mediated depletion of L-arginine [7], inducible nitric oxide synthase (iNOS) and NADPH oxidase (NOX2) production of reactive nitrogen and oxygen species [8,9], vascular endothelial growth factor (VEGF) over-expression [10], cysteine depletion [11], and the expansion of T-regulatory (Treg) cell populations [12,13]. While rare or absent in healthy individuals, MDSC accumulate in the settings of trauma, severe infection or sepsis, and cancer [6], possibly as a result of the hypoxia and inflammatory mediators in the tumor microenvironment [14-19]. In cancer patients and experimental tumor models, MDSC are major contributors to tumor immune tolerance and the failure of anti-tumor immunity [1]. Given the multitude of immune modulatory factors produced by tumors, it is indeed quite likely that different subsets of MDSC may be generated in the tumor microenvironment dependent upon the unique profile of factors secreted by the tumor [16,17,20]. Preclinical models of human tumor-induced MDSC will significantly advance knowledge of their induction and function as suppressor cells.

In a prior study, we demonstrated that certain cytokines can induce CD33^+ ^MDSC from normal donor peripheral mononuclear cells [16]. As an extension of these studies, we now report the development of a novel *in vitro *method to induce human MDSC from healthy donor peripheral blood mononuclear cells (PBMC) by co-culture with human solid tumor cell lines. Suppressor cells generated by this method demonstrate features consistent with MDSC isolated from cancer patients, including the inhibition of autologous T cell responses to stimuli [5]. Using this model system, we have determined the frequency of MDSC induction in human cancers of varied histiologic types, and have elucidated key tumor-derived factors that drive MDSC induction. Our methods generated highly purified human MDSC in quantities sufficient to enable robust morphology, phenotype, gene expression, and functional analyses. From these investigations two major subsets of MDSC have been identified that will help elucidate the role of these cells in the ontogeny, spread, and treatment of cancer.

## Methods

### Cell Lines and Cell Culture

Tumor cell lines were obtained from the American Type Culture Collection (ATCC) or were gifted to the Epstein laboratory. Tumor cell line authenticity was performed by cytogenetics and surface marker analysis performed at ATCC or in our laboratory. All cell lines were maintained at 37°C in complete medium [(RPMI-1640 with 10% fetal calf serum (characterized FCS, Hyclone, Inc., Logan, UT), 2 mM L-Glutamine, 100 U/mL Penicillin, and 100 μg/mL Streptomycin with 10 ng/mL hGM-CSF to support viability in co-cultures)], grown in tissue culture flasks in humidified, 5% CO_2 _incubators, and passaged 2-3 times per week by light trypsinization.

### Tumor-Associated MDSC Generation Protocol

#### i. Induction

Human PBMC were isolated from healthy volunteer donors by venipuncture (60 mL total volume), followed by differential density gradient centrifugation (Ficoll Hypaque, Sigma, St. Louis, MO). PBMC were cultured in complete medium (5-10 × 10^5 ^cells/mL) in T-25 culture flasks with human tumor cell lines for one week. Tumor cells were seeded to achieve confluence by day 7 (approximately 1:100 ratio with PBMC), and samples in which tumor cells overgrew were excluded from analysis and were repeated with adjusted ratios. Alternatively, irradiated tumor cells (3500 rad) were initially seeded at a 1:10 ratio in co-cultures to examine whether induction was dependent upon actively dividing tumor cells. PBMC cultured in medium alone were run in parallel as an induction negative control for each donor to control for any effects of FCS. For these studies 39 male and 22 female healthy, volunteer donors ages 23 to 62 were used under USC Institutional Review Board-approved protocol HS-06-00579. Data were derived from at least two individuals and no inter-donor differences in MDSC induction or function were observed.

For antibody neutralization experiments, PBMC-tumor cell line co-cultures were repeated in the presence or absence of neutralizing monoclonal antibodies for a subset of HNSCC cell lines and included anti-VEGF (Avastin, Genetech, San Francisco, CA), anti-TNFα (Humira, Abbott, Abbott Park, IL), anti-IL-1β (clone AB-206-NA, Abcam, Cambridge, MA), anti-IL-6 (clone AB-201-NA, Abcam), anti-GM-CSF (clone BVD2), anti-TGFβ (clone 1D11), anti-FLT3L (polyclonal, Abcam), or isotype control. For cytokine induction, PBMC were cultured at 5-10 × 10^5 ^cells/mL in complete medium supplemented with 10 ng/mL GM-CSF, FLT3L (25 ng/mL, Abcam), and/or TGFβ (2 ng/mL, R&D).

#### ii. MDSC Isolation

After one week, all cells were collected from tumor-PBMC co-cultures. Adherent cells were removed using the non-protease cell detachment solution Detachin (GenLantis, San Diego, CA). Myeloid cells were then isolated from the co-cultures using anti-CD33 or anti-CD11b magnetic microbeads and LS column separation (Miltenyi Biotec, Germany) as per manufacturer's instructions. Purity of isolated cell populations was found to be greater than 90% by flow cytometry and morphological examination and viability of isolated cells was confirmed using trypan blue dye exclusion.

#### iii. Suppression Assay

The suppressive function of tumor-educated myeloid cells was measured by their ability to inhibit the proliferation of autologous T cells in the following Suppression Assay: T cells isolated from 30 mL of PBMC from returning healthy donors by anti-CD8 microbeads and magnetic column separation (Miltenyi Biotec) were CFSE-labeled (3 μM, Sigma) and seeded in 96-well plates with myeloid cells isolated previously (*ii*. *MDSC isolation*, above) at 2 × 10^5^, cells/well 4:1 ratio. T cell proliferation was induce by anti-CD3/CD28 stimulation beads (Invitrogen, Carlsbad, CA). Suppression Assay wells were analyzed by flow cytometry for T cell proliferation after three days and supernatants were analyzed for IFNγ levels by ELISA (R&D Systems). Controls included a positive T cell proliferation control (T cells alone) and induction negative (medium only) and positive (GM-CSF + IL-6 cytokine-induced MDSC) controls [16]. Where indicated specific inhibitors of MDSC were added to suppression assays including all-*trans *retinoic acid (ATRA, 100 nM, Sigma, St. Louis, MO), sunitinib (0.1 μg/mL, ChemieTek, Indiannapolis, IN), celecoxib (15 μM, Pfizer, New York, NY), *nor-*NOHA (500 μM, CalBiochem, San Diego, Ca), L-NMMA (500 μM, Calbiochem), apocynin (0.1 mM, Sigma), 1D11 antibody (10 μg/mL), SB431542 (5 μM, Tocris, Ellisville, MO), or Avastin (10 μg/mL, Genentech, San Francisco, CA). Samples were run in duplicate and data were collected as percent proliferation for 15,000 cells. Samples were run on a FACSCalibur flow cytometer (BD Biosciences, San Jose, CA) and data acquisition and analysis were performed using CellQuestPro software (BD) at the USC Flow Cytometry core facility.

### Characterization of myeloid suppressor cells

#### i. Morphology of MDSC

Wright-Giemsa staining (Protocol Hema 3, Fisher, Kalamazoo, MI) of CD33^+ ^or CD11b^+ ^cell cytospin preparations was performed to assess the morphology of tumor-educated myeloid cells. Freshly isolated PBMC and CD33^+ ^cultured in medium only or induced by cytokines GM-CSF + IL-6 were prepared in parallel for comparison. Observation, evaluation, and image acquisition were performed using a Leica DM2500 microscope (Leica Microsystems, http://www.leica-microsystems.com) connected to an automated, digital SPOT RTke camera and SPOT Advanced Software (SPOT Diagnostic Instrument Inc., http://www.diaginc.com). Images were resized for publication using Adobe Photoshop software (Adobe, http://www.adobe.com).

#### ii. Flow cytometry analyses of cell phenotypes

The phenotype of *in vitro*-generated MDSC was examined for expression of myeloid, antigen-presenting, and suppressor cell markers. For staining, cells were collected from flasks using Detachin to minimize cell surface protein digestion, and washed twice with FACS buffer (2% FCS in PBS) before resuspending 10^6 ^cells in 100 μl FACS buffer. Cells were stained for 1hr on ice with cocktails of fluorescently-conjugated monoclonal antibodies or isotype-matched controls, washed twice with FACS buffer, and resuspended in FACS buffer for analysis. For intracellular staining, cells were fixed and permeabilized using Fixation/Permeabilization Kit (eBioscience, San Diego, CA) after surface staining. Antibodies used were purchased either from BD Biosciences: CD11c (B-ly6), CD33 (HIM3-4), HLA-DR (L243), CD11b (ICRF44), CD66b (G10F5), CD14 (M5E2), CD68 (Y1/82A), 41BBL (C65-485), OX40L (Ik-1); or eBioscience: CD30 (Ber-H2), CD103 (B-Ly7), GITRL (eBioAITR-L), CD56 (MEM-188). Samples were run on a BD FACSCalibur flow cytometer and data acquisition and analysis were performed as above. Data are from three unique donors and expressed as a fraction of labeled cells within a live-cell gate set for 15,000 events. CD33^+ ^or CD11b^+ ^cells from PBMC cultured in medium alone were run in parallel for comparison.

#### iii. Real-time RT-PCR for gene expression of myeloid suppressor cells and tumor cell lines

For gene expression studies, tumor-educated CD33^+ ^or CD11b^+ ^cells were isolated from tumor-PBMC co-cultures by fluorescence activated cell sorting after Induction (*i. Induction*, above) and RNA was isolated from MDSC and DNase-treated using Qiagen's RNeasy micro kit. Tumor cells were collected from culture flasks and RNA isolated and DNase-treated using Qiagen's RNeasy mini kit. For real-time RT-PCR, 100ng of DNase-treated RNA was amplified with gene specific primers using one-step Power SYBR green RNA-to-Ct kit (Applied Biosystems) and run in an MX3000P Strategene thermocycler (La Jolla, CA). Data were acquired and analyzed using MxPro software (Stratagene). Gene expression was normalized to housekeeping gene GAPDH and fold change determined relative to expression levels in medium only-cultured cells. Primer sequences were obtained from the NIH qRT-PCR database http://primerdepot.nci.nih.gov and were synthesized by the USC Microchemical Core Facility [21].

#### iv. Measurement of tumor-derived factors by ELISA

Supernatants were collected from confluent cell line cultures, passed through a 0.2 μm syringe filter unit to remove cell debris, and stored in aliquots at -20°C. Levels of IL-1β, IL-6, TNFα, VEGF, and GM-CSF in supernatant samples were measured using ELISA DuoSet kits (R&D) per manufacturer's instructions. Plate absorbance was read on an ELX-800 plate reader (Bio-Tek, Winooski, VT) and analyzed using KC Junior software (Bio-Tek).

#### v. Functional studies

Tumor cell line-induced CD33^+ ^or CD11b^+ ^MDSC and medium only controls were isolated by magnetic bead separation (Miltenyi Biotec) and used for functional studies. Arginase activity was measured in cell lysates using Bioassay Systems' QuantiChrom Arginase Assay Kit (Hayward, CA) per the manufacturer instructions. For measurement of ROS production, freshly isolated myeloid cells were incubated for 45 minutes in RPMI with 3 μM DCFDA (Sigma) then analyzed by flow-cytometry. Nitrites were measured in supernatants of cells cultured 5 × 10^5 ^cells/mL overnight in complete medium using Promega's Griess Reagent System (Madison, WI) per the manufacturer instructions.

#### vi. Immunohistochemistry

Immunohistochemistry studies were performed by the USC Department of Pathology Histology Core Facility (Los Angeles, CA) on cytospin preparations of suppressive and non-suppressive myeloid cells using antibodies against p-STAT3 (clone 6D779, dilution 1:400), C/EBPβ (clone H-7, dilution 1:100) (Santa Cruz Biotech), and HIF1a (clone 241812, dilution 1:50) (R&D Systems). Images were acquired and resized for publication as described above.

### Statistical analysis

Changes in mean T cell proliferation and mean IFNγ production in the presence or absence of tumor-educated or cytokine-treated MDSC were tested for statistical significance by one-way ANOVAs followed by Dunnett test for pairwise comparisons of experimental samples to T cells alone. Changes in mean T cell proliferation in suppression assays in the presence or absence of single inhibitors of suppressive mechanisms were evaluated by ANOVA followed by Tukey's test for pairwise comparisons between all groups. Mean gene expression of 15 tumor-derived factors between HNSCC cell lines with and without CD33^+ ^MDSC induction capacity was compared by ANOVA followed by Tukey's test for pairwise comparisons. For those factors with statistically significant different mean expression between suppressor cell inducing and non-inducing cell line groups, a linear regression analysis was performed to evaluate for a linear correlation between strength of suppressor cell induction and gene expression levels. Changes in mean T cell proliferation stimulated in the presence of suppressive CD33^+ ^or CD11b^+ ^cells induced by HNSCC or breast and lung carcinoma cell lines, respectively, for neutralization experiments were evaluated by ANOVA followed by Tukey's test for pairwise comparisons between all groups. Differences in mean expression of phenotypic markers between pooled groups of suppressive and non-suppressive CD33^+ ^or CD11b^+ ^cells were tested for significance by ANOVA followed by Bonferroni's multiple comparisons test for selected pairs (CD11b^+ ^MDSC vs. CD11b^+ ^medium control; CD33^+ ^MDSC vs. CD33^+ ^medium control). Differences in mean transcription factor or suppressive gene expression between CD11b^+ ^and CD33^+ ^MDSC were tested for significance by Student's t test. Differences in arginase activity, ROS production, and nitrite production among MDSC subsets and controls were evaluated by ANOVA followed by Bonferroni's multiple comparisons test for selected pairs (CD11b^+ ^MDSC vs. CD33^+ ^MDSC; CD11b^+ ^MDSC vs. CD11b^+ ^medium control; CD33^+ ^MDSC vs. CD33^+ ^medium control). Statistical tests were performed using GraphPad Prism software (La Jolla, CA) with a significance level of 0.05. Graphs and figures were produced using GraphPad Prism, Microsoft Excel, and Adobe Illustrator and Photoshop software (San Jose, CA).

## Results

### Induction of tumor-associated human myeloid suppressor cells

**Figure 1:**
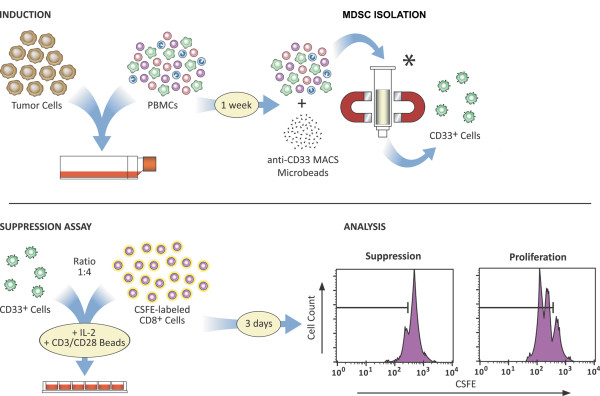
**Schematic of Co-culture and MDSC Suppression Assays for the *in vitro *generation of tumor-associated myeloid suppressor cells**. *Induction: *Normal donor PBMC are co-cultured with human solid tumor cell lines for one week. *MDSC Isolation: *CD33^+ ^or CD11b^+ ^cells are isolated from PBMC-tumor co-cultures by anti-CD33 or anti-CD11b microbead labeling and magnetic column separation. *Suppression Assay: *Tumor-educated CD33^+ ^or CD11b^+ ^cells are subsequently co-cultured with fresh, autologous CFSE-labeled T cells at a 1:4 ratio in the presence of T cell stimuli. After 3 days, T cell proliferation is measured as CFSE-dilution using flow cytometry. Suppressive function is evaluated as the ability of CD33^+ ^or CD11b^+ ^cells to inhibit autologous T cell proliferation.

A protocol for the generation of tumor cell line-educated human MDSC from normal donor PBMC was developed, as outlined schematically in Figure 1. Briefly, PBMC-tumor cell line co-cultures were established in tissue culture flasks for one week. Tumor-educated myeloid (CD33^+^) cells were then isolated, checked for viability, and tested for suppressive function by co-culture with fresh, autologous T cells in the presence of T cell stimuli. Use of irradiated tumor cells in co-cultures yielded comparable suppressor cell induction, suggesting that tumor cells need not be actively dividing to mediate the observed induction of suppressive function (Table 1). Unfractionated PBMC preparations were used in evaluating the ability of human solid tumor cell lines to generate myeloid suppressor cells to best approximate an *in vivo *setting, but CD33^+ ^suppressor cells were also generated successfully from T cell-depleted PBMC by co-culture with 4-998 osteogenic sarcoma or SCCL-MT1 head and neck squamous cell carcinoma (HNSCC) cells (Table 1).

**Table 1:** Canonical CD33^+ ^human MDSC induction by human cancer cell lines

Inducing Tumor Cell Line	Mean Percent Suppression	SEM	Inducing Tumor Cell Line	Mean Percent Suppression	SEM
**Controls**			**Cervical/Endometrial (4/5)**		

T cells alone	0.00		** HeLa	**68.35**	**5.36**
Medium only	-2.35	0.86	** ME-180	**75.24**	**3.83**
Lung Fibroblasts	-1.03	0.96	** SIHA	**54.49**	**8.66**
Ditt Fibroblasts	-0.13	2.91	** RL95-2	**52.11**	**3.84**
** GM-CSF + IL-6	**56.30**	**5.01**	SW 756	-83.60	2.18
**HNSCC (6/8)**			**Ovarian (6/9)**		

** SCCL-MT1	**91.83**	**0.82**	** A2780	**64.46**	**5.33**
*Irradiated*	**89.18**	**0.20**	** ES-2	**63.62**	**5.17**
*T cell Depl*.	**81.49**	**4.98**	** TOV-21G	**52.86**	**11.37**
** USC-HN2^1^	**87.97**	**ND**	** SK-OV-3	**51.44**	**9.81**
** SCC-4	**65.72**	**2.08**	* NIHOVCAR-3	**47.89**	**1.08**
** CAL-27	**66.26**	**6.21**	* SW 626	**46.54**	**4.07**
** SW 451	**59.49**	**9.59**	HOC-7	41.77	19.15
* FaDu	**30.98**	**4.45**	HEY	22.20	3.87
RPMI 2650	17.46	5.01	Caov-3	-146.53	2.69
SW 2224	-13.48	11.21	**Breast (0/9)**		
			
**Thyroid (1/2)**			MCF-7	16.95	0.39
			
** SW 579	**68.97**	**3.41**	734B	16.72	2.32
SW 1949	43.90	13.68	T47D	8.47	1.23
**Brain (2/9)**			BT-474	0.83	11.53
			
** NU-04	**69.41**	**4.02**	SKBR3	-0.09	13.53
** U118MG	**51.96**	**1.48**	MDA-MB-468	-3.46	0.25
SW 598	14.29	4.14	GI-101	-6.41	0.92
A172	2.26	4.97	SV-BR-1	-8.00	1.75
IMR-5	-1.23	3.09	MDA231	-16.21	2.60
IMR-32	-3.16	7.48	**Bladder (1/3)**		
			
TE 671	-12.23	4.29	** T24	**53.89**	**3.97**
Y79	-72.63	5.58	SW 780	8.10	10.01
BM-166	-83.22	0.05	SW 733	-54.63	0.45
**Melanoma (1/3)**			**Prostate (2/3)**		

** A375	**56.16**	**0.64**	** DU 145	**54.73**	**2.07**
CaCl74-36	17.26	6.83	* LNCaP	**29.09**	**2.78**
Colo 38	15.83	1.49	PC3	15.12	9.09
**Sarcomas (4/9)**			**Renal (3/6)**		

** 4-998	**58.31**	**0.82**	** 786-O	**75.91**	**6.06**
*Irradiated*	**52.10**	**0.44**	** CAKI-1	**64.94**	**3.70**
*T cell Depl*.	**65.23**	**8.17**	** CAKI-2	**63.62**	**5.17**
* Rh30	**44.63**	**2.51**	SW 156	36.51	10.69
* HOS	**42.58**	**4.86**	ACHN	9.85	0.20
* SW 1353	**42.22**	**4.42**	SK-NEP-1	0.00	1.82
HT 1080	19.37	5.92	**Gastric (1/2)**		
			
SA-4	12.53	1.05	** SW 1961	**64.55**	**3.04**
HS 919	3.01	5.31	KATO-III	7.65	2.16
SW 80	-5.00	3.93	**Colorectal (5/6)**		
			
HS 913T	-56.35	1.45	** SW 732	**69.19**	**1.29**
**Lung (4/11)**			** DLD-1	**65.59**	**3.19**
**Non-small cell (2/7)**			** SW 608	**53.11**	**5.15**
			
* A427	**27.71**	**6.87**	** SW 707	**52.38**	**0.64**
* SW 1573	**21.47**	**1.64**	* HT-29	**38.37**	**4.91**
NCI-H292	8.23	2.89	LS147T	13.62	3.87
NCI-H1650	6.67	4.18	**Pancreatic (3/10)**		
			
SK-MES-1	4.31	6.03	** SW 1990	**78.15**	**1.21**
NCI-H125	1.54	3.69	* Panc 2.03	**22.28**	**4.37**
NCI-H1975	0.28	2.89	* Panc 4.14	**21.82**	**2.60**
**Small Cell 2/3)**			Panc 9.6.94	27.28	8.47
			
** NCI-H464	**63.96**	**6.00**	Panc-1	7.82	3.69
* NCI-H60	**47.79**	**7.71**	Panc 3.27	6.98	5.44
NCI-HUT 69C	-24.28	16.75	ASPC-1	3.09	2.36
**Mesothelioma (0/1)**			CAPAN-1	-1.34	2.27
			
SW 1503	1.93	2.02	Panc 2.5	-1.79	6.08
**Liver (2/5)**			MIA PaCa-2	-4.38	1.94
			
* HA 22T	**44.01**	**4.22**	**Epidermoid (0/1)**		
			
* HEP 3B	**23.52**	**7.44**	A431	-31.12	8.55
PLC	22.05	5.22			
MAH	11.87	4.72			
HEP-G2	-2.10	9.40			

Forty-five of 101 human solid tumor cell lines induce functionally suppressive CD33^+ ^myeloid suppressor cells from volunteer normal human PBMC after one-week co-culture *in vitro*. Tumor cell lines inducing CD33^+ ^MDSC with statistically significant suppressive function are indicated by */bold, and those with strong MDSC inducing capacity (mean T cell suppression by CD33^+ ^cells ≥ 50%) are indicated by **. CD33^+ ^cells from PBMC cultured in complete medium alone (non-suppressive control), co-cultured with fibroblast cell lines (induction negative control), and cytokine-induced MDSC (GM-CSF + IL-6, suppressive control) were run in parallel for comparison. Irradiated tumor cell lines and T cell depleted PBMC (italicized) were tested for the ability to induce CD33^+ ^MDSC in some experiments.

### Strong CD33^+ ^MDSC induction capability by a subset of human tumor cell lines

MDSC have been reported in patients with a wide range of different types of cancer [21-31] and their accumulation appears to correlate with increased tumor burden and stage [10,30]. However, it remains unclear whether all cancers induce this tolerizing population, as strong evidence exists to suggest diversity in immune escape mechanisms amongst cancer types and individual tumors [32]. To address this question, one-hundred-one human solid tumor cell lines were tested for their ability to induce MDSC in the tumor co-culture assay using PBMC from 61 unique healthy, volunteer donors (39 male, 22 female) ranging in age from 23-62 (Table 1). CD33^+ ^MDSC could be generated by at least one cell line of every human tumor type examined (cervical/endometrial, ovarian, pancreatic, lung, head and neck, renal cell, liver, colorectal, prostate, thyroid, gastric, bladder, sarcoma, and glioblastoma), with the exception of breast carcinoma (Table 1). Head and neck, cervical/ovarian, colorectal, and renal cell carcinoma cell lines frequently induced CD33^+ ^MDSC and are good models for further studies of this suppressive population. A range of suppressor cell ability appeared to exist within histologic types for the majority of tumor cell lines examined, suggesting that subclones within a whole tumor may drive MDSC induction. Notably, myeloid cells from PBMC cultured in medium alone or co-cultured with fibroblast cell lines were not suppressive (Table 1).

### Tumor cell line-induced CD33^+ ^MDSC resemble MDSC from cancer patients in suppressive function and gene expression

**Figure 2:**
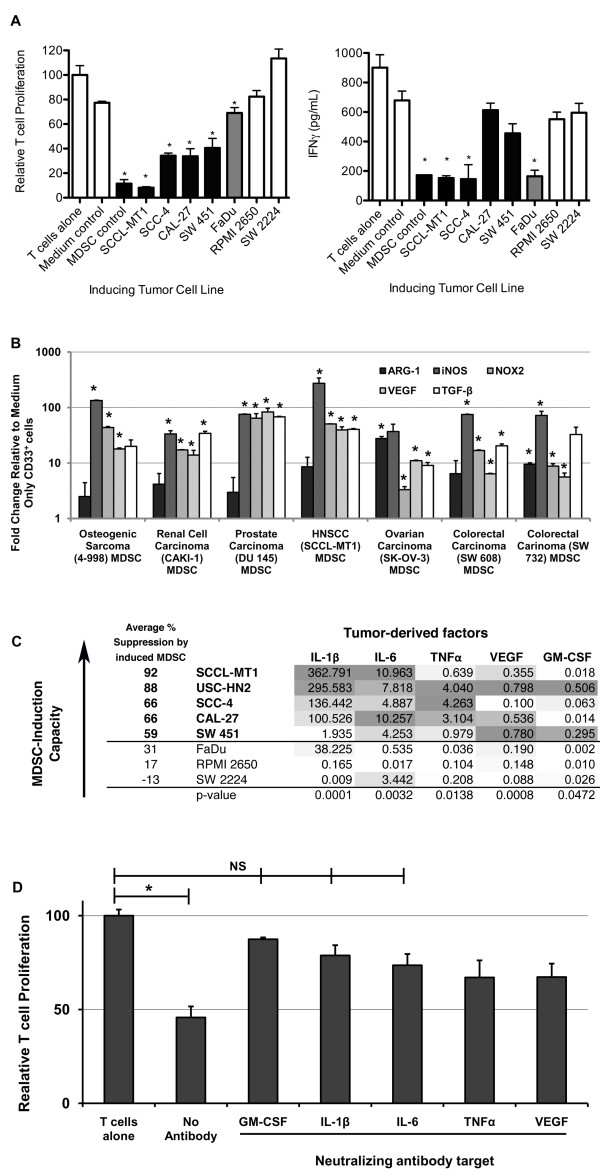
**Induction and functional characterization of canonical CD33^+ ^MDSC by human tumor cell lines**. *A*, HNSCC-induced MDSC inhibit autologous T cell proliferation and IFNγ production. A subset of HNSCC cell lines induces a CD33^+ ^population with suppressive function characteristic of MDSC, including inhibition of autologous T cell proliferation (*left*) and IFNγ secretion (*right*). Tumor cell lines are grouped by strength of MDSC induction: strong (black), weak (gray), and non-inducing (white). For both graphs, mean shown (n ≥ 2 donors) +SEM. * indicates statistical significance by ANOVA followed by Dunnett post-test for comparison to T cells alone, p <0.05. *B*, Human MDSC mediate suppression through up-regulation of ARG-1, NOX2, iNOS, VEGF, and TGFβ. *A*, Expression of putative suppressive genes ARG-1, iNOS, NOX2-component NCF1, VEGF, and TGFβ in a subset of tumor cell line-induced CD33^+ ^MDSC. Mean fold change (n ≥ 2 donors per tumor cell line) +SEM, relative to CD33^+ ^cells cultured in medium alone, are shown. * indicates statistical significance, p <0.05, by ANOVA followed by Dunnett test for pairwise comparisons to medium only CD33^+ ^controls. *C*, Heatmap showing expression of immune modulatory cytokines by HNSCC cell lines in relation to their ability to induce CD33^+ ^MDSC. MDSC-inducing cell lines produce increased IL-1β, IL-6, TNFα, VEGF, and GM-CSF. Expression of ten putative MDSC-inducing factors was measured in MDSC-inducing (bold) and non-inducing HNSCC cell lines by qRT-PCR. Increased CD33^+ ^MDSC-induction capacity was associated with greater expression of IL-1β, IL-6, TNFα, and VEGF (p <0.05). Mean fold change (n = 2) relative to human reference RNA (gray shading = increased, white = decreased expression), p value shown is for linear regression analysis for factors having significantly higher gene expression in MDSC-inducing compared with non-inducing human HNSCC cell lines by one-way ANOVA followed by Tukey's post-test. *D*, Removal of GM-CSF, IL-6, or IL-1β from co-culture impairs CD33^+ ^MDSC induction by tumor cell lines. T cell proliferation when co-cultured with CD33^+ ^MDSC from tumor cell line (SCCL-MT1 or USC-HN2) co-cultures with neutralizing antibodies to GM-CSF, IL-6, IL-1β, TNFα, or VEGF. Mean shown (n = 5, four independent experiments), +SEM. * indicates statistical significance, p <0.05.

A sample of HNSCC cell line-induced CD33^+ ^MDSC (from co-cultures with SCCL-MT1, SCC-4, CAL-27, FaDu, RPMI 2650, or SW 2224) were used to characterize further the suppressive function and related gene expression of these *in vitro*-generated suppressor cells. As shown in Figure 2A, tumor cell line-educated MDSC suppressed both autologous T cell proliferation and interferon γ with a range of suppressive function seen amongst MDSC samples induced by different HNSCC cell lines. The suppressive capability of HNSCC-induced MDSC was compared with that of a positive T cell proliferation control (T cells alone), an induction negative control (CD33^+ ^cells from medium only cultures), and an induction positive control (CD33^+ ^cells isolated from PBMC cultured with GM-CSF and IL-6). Of note, while the most potent MDSC (SCCL-MT1 and SCC-4-induced) blocked both T cell proliferation and IFNγ production, weaker HNSCC-induced CD33^+ ^suppressor cells preferentially inhibited T cell proliferation (CAL-27 or SW 451-induced) or IFNγ production (FaDu-induced). These findings suggest that MDSC may impede T cell responses through multiple avenues, including inhibition of activation and expansion.

**Figure 3:**
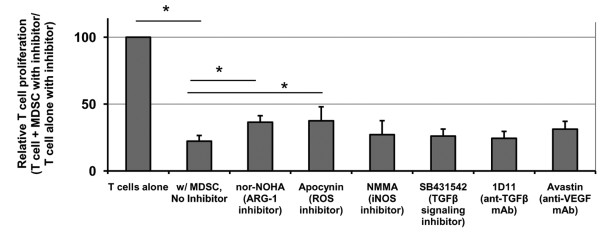
**Tumor cell line-induced CD33^+ ^MDSC inhibit proliferation of autologous, CD3/CD28-stimulated T cells through multiple mechanisms**. Specific inhibitors of MDSC suppressive mechanisms ARG-1 and NOX2 mediate partial but incomplete reversal of suppression. * indicates statistical significant difference in mean T cell proliferation (mean shown + SEM, n ≥ 7 for each inhibitor, data from 2 independent experiments with similar results), p <0.05, by ANOVA followed by Tukey test for pairwise comparisons.

Using these and additional tumor cell line-induced MDSC samples (4-998 osteogenic sarcoma, DU 145 prostate carcinoma, CAKI-1 renal cell carcinoma, SK-OV-3 ovarian carcinoma, and SW 608 and SW 732 colorectal adenocarcinoma cell lines), we analyzed expression of putative MDSC suppression genes in comparison to normal myeloid cells. These MDSC consistently showed statistically significant up-regulation of ARG-1, iNOS, NOX2, VEGF, and/or TGFβ compared with control CD33^+ ^cells from medium-only cultures (Figure 2B). Subtle variations were observed in the gene expression patterns of these tumor-induced MDSC, which is consistent with the hypothesis that different MDSC subsets are generated by different tumors dependent upon the specific profile of immune factors produced by each. To determine the dominant mechanism of T cell suppression by this canonical CD33^+ ^MDSC subset, suppression assays were repeated in the presence or absence of specific inhibitors of ARG-1 (nor-NOHA), iNOS (L-NMMA), NOX2 (apocynin), VEGF (neutralizing antibody Avastin), or TGFβ1 (SB431542 or neutralizing antibody 1D11). In these studies no one inhibitor was found to completely reverse suppression (Figure 3), consistent with the pleotropic actions of MDSC, but inhibitors of ARG-1 and NOX2 did produce statistically significant decreases in suppression by CD33^+ ^MDSC. These results were confirmed by siRNA knockdown of individual suppression genes: ARG-1, iNOS, NCF1 (NOX2 component), TGFβ1, or VEGFA (data not shown).

### CD33^+ ^MDSC are induced by tumor-derived IL-1β, IL-6, TNFα, VEGF, and GM-CSF

**Figure 4:**
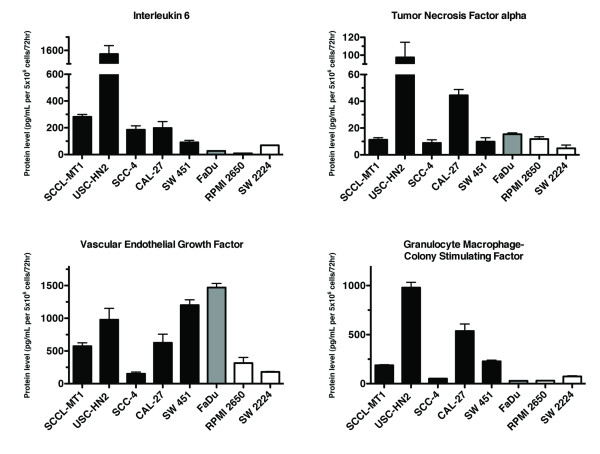
**MDSC-inducing cell lines produce increased GM-CSF, IL-6, TNFα, and VEGF**. Protein secretion of these cytokines by HNSCC cell lines was measured in supernatants using ELISA techniques to confirm gene expression findings. Mean protein levels shown (two independent experiments each run in triplicate), +SEM. Of note, cell line USC-HN2 was recently established and characterized in our laboratory from a the tumor of a patient with recurrent oral cavity squamous cell carcinoma^1^ and found to be a strong producer of immune modulatory factors associated with MDSC induction.

Previously, we compared gene expression of immune modulatory cytokines for groups of MDSC-inducing and non-inducing human cancer cell lines [16]. These studies suggested multiple mechanisms of MDSC induction amongst tumor cell lines, including inflammatory cytokines. To reduce background differences in gene expression related to tissue-specific expression patterns, a group of human HNSCC cell lines consisting of both MDSC-inducing and non-inducing models was further studied for expression of these putative MDSC inducing factors. HNSCC tumor cell lines showed a high frequency of CD33^+ ^MDSC induction (Table 1) and thus were good models for further studies of induction. Expression of immune modulatory factors (c-kitL, COX2, FLT3L, GM-CSF, IL-1β, IL-4, IL-6, IL-10, IDO, iNOS, M-CSF, TGFβ, TNFα, VEGF) was measured in eight HNSCC cell lines using quantitative RT-PCR techniques. As shown in Figure 2C, MDSC-induction capacity correlated directly with tumor cell line expression of IL-1β, IL-6, TNFα, VEGF, and GM-CSF (p <0.05 for ANOVA followed by Dunnett test for pairwise comparisons between inducing and non-inducing cell lines for each factor, and p <0.05 for linear regression analysis of suppressive induction capacity with level of cytokine production). Differential gene expression of IL-6, TNFα, VEGF, and GM-CSF was confirmed at the protein level by ELISA techniques (Figure 4); IL-1β levels were below the sensitivity of the assay. These data concur with our previous work showing that IL-6, IL-1β, VEGF, and TNFα with GM-CSF are sufficient for CD33^+ ^MDSC induction from normal donor PBMC [16]. Neutralizing antibodies to cytokines GM-CSF, IL-1β, IL-6, VEGF, or TNFα were tested in PBMC-tumor cell line co-cultures to determine which factor(s) was most important for induction (Figure 2D). Neutralization of GM-CSF, IL-6, or IL-1β in tumor cell line-PBMC co-cultures abrogated significant induction of CD33^+ ^suppressor cell function (p <0.05, significant differences between these conditions and induction without neutralizing antibodies) and restored T cell proliferation to levels comparable to controls (p = NS). COX2 expression was also elevated in many of the MDSC-inducing cell lines, particularly ovarian and cervical cancer cell lines, and PGE_2 _in combination with GM-CSF induced weak suppressive function in CD33^+ ^cells ([16], data not shown). However, addition of COX2 inhibitors to ovarian and cervical tumor cell line-PBMC co-cultures did not significantly decrease MDSC induction (data not shown).

### Preferential induction of a second subset of CD11b^+ ^MDSC by some human cancer cell lines through FLT3L and TGFβ

**Figure 5:**
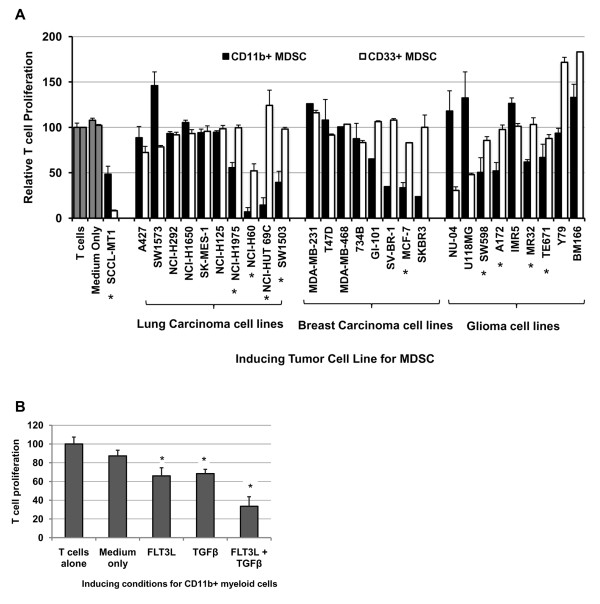
**Induction of a second CD11b^+ ^MDSC subset by breast, lung, and brain cancer cell lines**. *A*, CD11b^+ ^cells from breast cancer, lung cancer, or glioma cell line-PBMC co-cultures were evaluated for suppressive function against CD3/CD28 stimulated autologous T cells. Mean (n = 2) T cell proliferation + SEM or T cell proliferation (n = 1) is shown from Suppression Assays of CD33^+ ^or CD11b^+ ^cells with autologous T cells, respectively. * indicates statistically significant suppression of T cells by CD11b^+ ^cells from co-culture (p <0.05, ANOVA followed by Dunnett's test for comparison to T cells alone); significance for suppression by CD33^+ ^cells is found in Table 1. Note that some tumor cell lines induce both subsets, while others induce only one subset or neither. CD33^+ ^and CD11b^+ ^cells from medium only cultures were not suppressive. *B*, CD11b^+ ^MDSC subset can be induced from normal donor PBMC by cytokines FLT3L and TGFβ. Mean shown (n = 3) + SEM. * indicates statistically significant in mean T cell proliferation compared with T cells alone (p <0.05).

Interestingly, no human breast cancer cell line (0/9) tested generated CD33^+ ^MDSC from PBMC after a one-week co-culture (Table 1). This finding led us to investigate the induction of other MDSC phenotypes by these models. Human MDSC have been reported to express a wide range of surface markers and likely consist of several subtypes [2,5,20,22,24,27,29,30]. In addition to the common myeloid antigen CD33, CD11b is another marker reported to be expressed on some human MDSC [3,5,33]. As shown in Figure 5A, breast carcinoma cell lines preferentially induced CD11b^+ ^MDSC, suggesting that this component of the MAC-1 phagocytic complex may be a more specific marker for the subset of MDSC induced by this tumor type. Lung carcinoma and glioma cell lines, which had a low frequency of CD33^+ ^MDSC induction, also were found to induce with moderate frequency the CD11b^+ ^MDSC subset (Figure 5A). Taken collectively with our survey of CD33^+ ^MDSC induction, these data suggest that the induction of MDSC is a universal feature of human cancers with some variation in the phenotype of induced MDSC subsets observed. These data further emphasize the importance of functionally defining this heterogeneous population of suppressor cells until specific activation-associated markers are identified.

Revisiting previously published gene expression data for this group of breast cancer cell lines, which lack CD33^+ ^MDSC induction, we identified FLT3L and TGFβ as differentially expressed candidates for CD11b^+ ^MDSC subset induction from our panel of putative MDSC-inducing factors [16]. PBMC were then cultured in the presence of FLT3L, TGFβ, FLT3L + TGFβ, or medium alone for one week to evaluate whether these cytokines were sufficient for CD11b^+ ^MDSC induction. Myeloid cells isolated from cytokine-treated cultures showed significant suppression of autologous T cell proliferation (p <0.05, comparison to T cells cultured alone), consistent with MDSC, with the most potent cells generated from combined FLT3L and TGFβ treatment (Figure 5B). These data suggest that FLT3L and TGFβ are present and sufficient for CD11b^+ ^MDSC induction, but technical difficulties in abolishing FLT3L, which is a broad hematopoietic progenitor growth factor, and TGFβ, which is ubiquitous in serum and regulated by association of a latency protein, precluded clear neutralization data.

### Characterization of human CD33^+ ^and CD11b^+ ^suppressor cells induced by tumor cell lines

**Figure 6:**
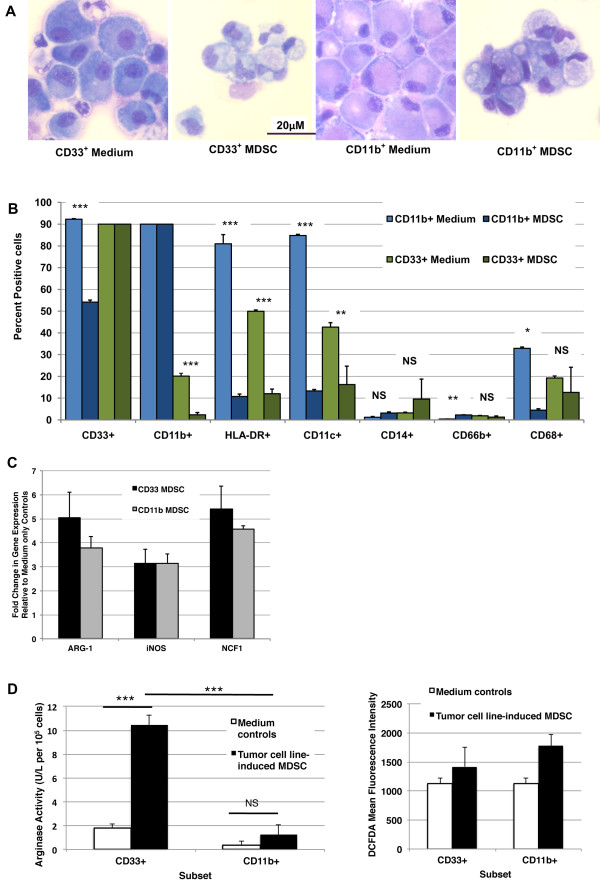
**Morphology, phenotype, and function of CD11b^+ ^and CD33^+ ^MDSC subsets**. *A*, Morphology of human CD33^+ ^and CD11b^+ ^MDSC subsets isolated after tumor cell line co-culture and normal myeloid counterparts from medium only cultures (Wright-Giemsa staining, x400, original magnification). CD33^+ ^MDSC appear slightly more differentiated than CD11b^+ ^MDSC after induction. Images are representative of data from more than five donors and three independent experiments using SCCL-MT1 or USC-HN2 for CD33^+ ^MDSC induction and MCF7 or NCI-H60 for CD11b^+ ^MDSC induction. *B*, Human CD33^+ ^and CD11b^+ ^MDSC are distinct subsets with a common HLA-DR^low ^Lineage^- ^phenotype. Phenotype of HNSCC cell line-induced CD33^+ ^and breast cancer cell line-induced CD11b^+ ^MDSC compared with medium only, non-suppressive CD33^+ ^and CD11b^+ ^cells as measured by flow cytometry. Mean percent positive cells (n ≥ 2) + SD shown, data from three unique donors. Differences in percent positive cells analyzed by ANOVA then Bonferroni's multiple comparison test for selected pairs (* indicates statistically significant difference in mean percent positive between MDSC and medium control for each subset, p <0.05). *C*, Comparison of ARG-1, iNOS, and NOX2-component NCF1 gene expression in CD33^+ ^and CD11b^+ ^human MDSC revealed similar levels of expression between these subsets. Mean fold change shown relative to medium only controls (n = 3 unique donors for MDSC from co-cultures with each of three inducing tumor models) + SEM. No statistically significant difference between means as determined by Student's t test for each gene. *D*, Elevated arginase activity (left) and reactive oxygen species (ROS) production (right) by tumor cell line-induced CD33^+ ^and CD11b^+ ^MDSC. Arginase activity of CD11b^+ ^and CD33^+ ^MDSC subsets as measured by arginine degradation to urea and compared with normal myeloid cells. Mean shown + SEM; data from four unique donors and two inducing cancer cell lines for each subset. * indicates statistical significance (p <0.05); NS = not significant. ROS production by CD11b^+ ^and CD33^+ ^MDSC subsets as measured by DCFDA and compared with normal myeloid cells. Mean fluorescence intensity shown + SEM for 20,000 events collected; data from three unique donors and two inducing cancer cell lines for each subset. No significant difference by ANOVA.

To characterize better these two MDSC subsets (CD11b^+ ^or CD33^+^), comparative morphology, phenotype, gene expression, and functional studies were performed. The morphology of suppressive tumor-co-cultured CD33^+ ^and CD11b^+ ^populations was compared to that of freshly isolated PBMC and myeloid cells cultured in medium only by Wright-Giemsa staining (Figure 6A and data not shown). Healthy donor PBMC showed occasional mononuclear cells with pale and scant cytoplasm, scattered amongst predominant lymphocytes (data not shown). CD33^+ ^and CD11b^+ ^cells from PBMC cultured in medium alone (with hGM-CSF for growth support) for one week were predominantly large, mononuclear cells having abundant basophilic cytoplasm with occasional granulocytes (CD33^+ ^only) and other myeloid lineage cells (*e.g*. eosinophils) (*far left and middle right panels*). In contrast to the mature lineages seen in medium only myeloid cells, CD33^+ ^and CD11b^+ ^suppressor cells isolated from PBMC after tumor co-culture (USC-HN2 or SCCL-MT1 HNSCC for CD33^+^, MCF7 breast or NCI-H60 small cell lung for CD11b^+^) showed an abundance of immature cells, including metamyelocytes or band cells and blast-like cells (*middle left and far right panels, representative images shown of three independent experiments*). Subtle morphologic differences were observed between CD33^+ ^and CD11b^+ ^MDSC, which pointed to the fact that CD11b^+ ^MDSC appeared more immature than CD33^+ ^suppressor cells (Figure 6).

### Phenotype of MDSC shows CD33^+ ^and CD11b^+ ^subsets to be both HLA-DR^low ^and Lineage^-^

**Figure 7:**
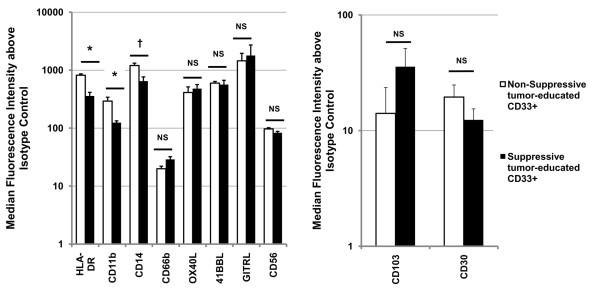
**Expanded phenotype of canonical CD33^+ ^human MDSC subset induced by tumor cell lines**. Phenotype Expression of antigen presenting cell (left) and suppressor cell (right) markers on strongly suppressive (induced by HNSCC cell lines SCCL-MT1, SCC-4, CAL-27) versus non-suppressive (induced by SW 2224, RPMI 2650, or medium only) CD33^+ ^myeloid cells as measured by flow cytometry. Median fluorescence above isotype control (data from 3 unique donors; mean shown for all three induction conditions (n = 9) +SEM). * indicates statistical significance, p <0.05, † indicates p = 0.59 for comparisons between suppressive cell and non-suppressive cells mean.

**Figure 8:**
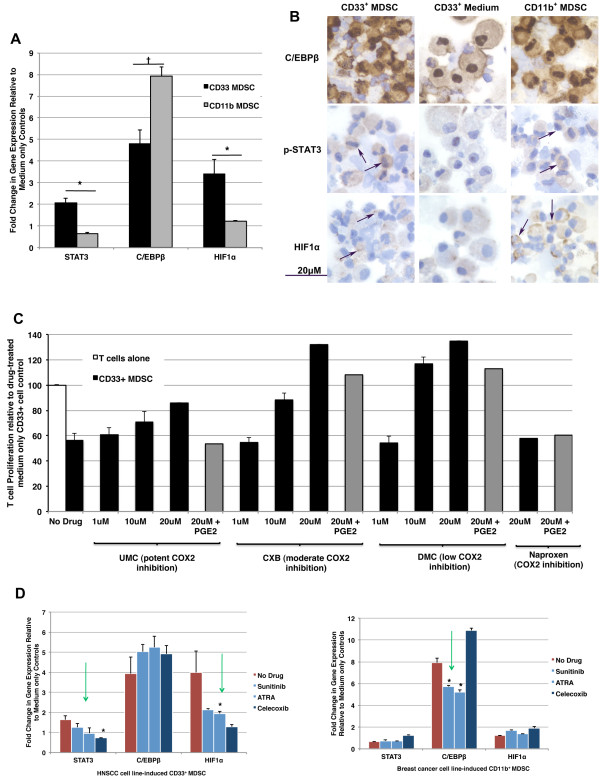
**Transcription factors promoting human MDSC suppressive function**. *A*, HIF1α, STAT3, and C/EBPβ expression in tumor cell line-induced CD33^+ ^or CD11b^+ ^MDSC compared with medium only controls as measured by qRT-PCR. Mean shown (data from six unique donors, two independent experiments) +SEM; * indicates statistical significance, p <0.05, † indicates p = 0.06. *B*, Immunohistochemisty of C/EBPβ, p-STAT3, and HIF1α in CD33^+ ^(*left*) and CD11b^+ ^(*right*) MDSC and CD33^+ ^medium controls (*middle*). Representative images shown from multiple samples stained (400x, original magnification) with arrows showing positive staining areas for p-STAT3 and HIF1α. *C*, Inhibition of CD33^+ ^human MDSC subset by celecoxib and celecoxib analogs via a non-COX2 dependent mechanism. Studies in our laboratory have identified Celecoxib and analogs dimethyl celecoxib (DMC) and unmethylated celecoxib (UMC) as inhibitors of suppressive function in CD33^+ ^MDSC *in vitro*. Of note, the reversal of MDSC effects by CXB and analogs DMX and UMC does not appear to rely upon cyclo-oxygenase (COX)2 enzyme inactivation, as demonstrated by the persistence of therapeutic effects in the presence of prostaglandin E_2 _rescue, efficacy of analog DMC with low to absent COX inhibitory action, and the absence of effect seen with the structurally-unrelated COX2-seletcive inhibitor naproxen. For these studies, human CD33^+ ^MDSC induced by cancer cell lines were co-cultured with fresh, autologous CFSE-labeled T cells at a ratio of 1:4 in the presence or absence of drugs (black bars) and prostaglandin E2 (PGE2, gray bars) as indicated. T cell stimulation was provided by anti CD3/CD28 microbeads. After three days in culture, T cell proliferation was measured as CFSE dilution by flow cytometry. Mean T cell proliferation shown where possible (n = 2 for no drug, 1 μM, and 10 μM; n = 1 for 20 μM and 20 μM + PGE2) + SD, two independent experiments. *D*, Transcriptional changes in MDSC subsets associated with inactivation of suppressive function. (*left panel*) Reversal of CD33^+ ^MDSC suppressive function by ATRA, sunitinib, and CXB correlated with decreased STAT3 and HIF1α expression (green arrows). (*right panel*) Functional inhibition of human CD11b^+ ^MDSC by ATRA and Sunitinib correlated with decreased C/EBPβ levels (green arrow), but no change in STAT3 and HIF1α mRNA levels. CXB was not found to have inhibitory actions on CD11b^+ ^MDSC and it was not observed to decrease C/EBPβ levels in this population. Mean shown (data from three unique donors) + SEM, * indicates statistically significant decrease (p <0.05) in transcript level in drug-treated MDSC compared with untreated MDSC (ANOVA with Dunnett post-test).

Further characterization of CD33^+ ^and CD11b^+ ^MDSC subsets was performed using a wide range of proposed MDSC and mature innate immune cell markers (CD33, CD11b, CD66b, CD14, CD11c, HLA-DR, GITRL, OX40L, 41BBL (CD137L), CD56). Human MDSC were isolated by magnetic bead column separation after one-week co-culture with SCCL-MT1 or USC-HN2 HNSCC cell lines (CD33^+^) or MCF-7 breast cancer cell line (CD11b^+^) and non-suppressive CD33^+ ^or CD11b^+ ^control cells were isolated from medium only PBMC cultures. The purity for column isolated populations was found to be >90% by flow cytometry. Positivity for MDSC and mature myeloid lineage markers was measured by flow cytometry for each population and compared between CD33^+ ^and CD11b^+ ^MDSC subsets and between suppressive and non-suppressive populations (Figure 6B). Interestingly, CD11b expression levels were inversely correlated with suppressive function in CD33^+ ^cells in these studies, and similarly CD33 positivity was inversely correlated with suppressive function in CD11b^+ ^cells, suggesting a divergence in the two populations during induction. Notably, both CD33^+ ^and CD11b^+ ^suppressive populations showed decreased expression of activation marker HLA-DR and mature dendritic cell (DC) marker CD11c compared with non-suppressive populations of CD11b^+ ^and CD33^+ ^cells. These data are consistent with an accumulation of immature myeloid lineage cells coincident with the induction of suppressive function in either CD11b^+ ^or CD33^+ ^cells. Differentiated DC markers and T cell co-stimulatory ligands were further examined on the CD33^+ ^subset of MDSC and found to be expressed at similarly low levels between suppressive and non-suppressive CD33^+ ^cells isolated from tumor co-cultures (p = NS) (Figure 7), suggesting that the maturation and antigen presenting defects of MDSC are not primary in T cell suppression. This is consistent with therapeutic studies we have performed in our laboratory in which the addition of T cell co-stimulatory ligands (Fc-huGITRL, Fc-huCD137L, Fc-B7.1) or agonist antibodies (anti-huCD137, anti-huGITR, anti-huCD28) to suppression assays failed to significantly reverse inhibition of T cell proliferation (p = NS) (data not shown). Two surface markers, CD30 and CD103, found on other immune suppressor cell populations [34,35] were examined in this study as potential unique markers of active MDSC, but were not found to correlate with their suppressive function (p = NS) (Figure 8). Macrophage marker CD68 and granulocyte marker CD66b expression were low or absent and not differentially expressed by suppressive and non-suppressive CD33^+ ^or CD11b^+ ^cells in this study, emphasizing that that these phenotypes likely do not represent tumor-associated macrophages [1] or the granulocytic MDSC subsets described elsewhere [36].

### Comparison of suppressive function in CD33^+ ^and CD11b^+ ^MDSC subsets

A comparison of ARG-1, iNOS, and NOX2-component NCF1 gene expression in CD33^+ ^and CD11b^+ ^human MDSC induced by HNSCC or breast and lung carcinoma cell lines, respectively, revealed similar levels of expression between these subsets with a trend toward increased ARG-1 and NOX2 expression in CD33^+ ^MDSC (Figure 6C). Functional studies confirmed greater arginase activity in CD33^+ ^versus CD11b^+ ^MDSC, but suggested that reactive oxygen species production is similarly elevated in both subsets (Figure 6D). Nitrite production was not found to be greatly elevated above medium only controls (data not shown), perhaps indicating that iNOS activity is a minor contributor for suppressive function in these subsets. While these findings remain preliminary, they suggest partial or complete functional overlap of these MDSC subsets. Furthermore, these data suggest that effective abrogation of human MDSC activities by depletion of a single subset is unlikely to yield significant therapeutic benefit in cancer patients that induce both subsets.

### Higher Hif1α, STAT3, and C/EBPβ gene expression delineate subsets and distinguish tumor cell line-induced human MDSC from normal myeloid cells

It is apparent that human MDSC can be induced by multiple factors present in the tumor microenvironment [16]. Furthermore, as a consequence of these multiple different induction routes, at least two distinct phenotypes of human MDSC emerge that can both mediate suppression of T cell responses. Interestingly, these CD33^+ ^and CD11b^+ ^MDSC subsets showed some phenotypic (HLA-DR^low ^and lineage^-^) and functional convergence despite preferential induction by different tumor models and predominant expression of either CD33 or CD11b. We wondered whether a common transcription factor was activated by these multiple pathways and might be act as a "master switch" to control both of these human MDSC. Several transcription factors have been proposed for control of MDSC, primarily in mice, including CCAAT-enhancer-binding proteins (C/EBP) β [37], hypoxia inducible factor (HIF) 1α [14], and signal transducer and activator of transcription (STAT) 3 [26,38], STAT5 [38], and STAT6 [2]. Previously identified as transcriptional regulators in some murine tumor-derived MDSC subsets, we now show that these transcription factors are elevated in human MDSC and, importantly, are differentially expressed in CD33^+ ^versus CD11b^+ ^MDSC subsets. We examined the expression of HIF1α, STAT3, and C/EBPβ in tumor cell line (SCCL-MT1 or USC-HN2)-induced CD33^+ ^or (MCF7 breast or NCI-H60 small cell lung carcinoma) CD11b^+ ^human suppressor cells compared with medium only controls by qRT-PCR techniques (data from six unique donors, two independent experiments) (Figure 8A) and immunohistochemistry (Figure 8B). Both CD33^+ ^and CD11b^+ ^functionally active human MDSC showed significant up-regulation of transcription factors STAT3, C/EBPβ, and HIF1α compared with non-suppressive myeloid cells from medium only cultures. However, CD33^+ ^and CD11b^+ ^MDSC subsets showed differences in transcriptional changes for these factors that were suggestive of different induction or activation pathways. As shown previously, CD33^+ ^or CD11b^+ ^MDSC may be induced under a variety of different tumor conditions and following incubation with several distinct cytokine mixtures [16]. CD33^+ ^MDSC showed stronger up-regulation of STAT3 and HIF1α while CD11b^+ ^MDSC showed comparably greater up-regulation of C/EBPβ (Figure 8A). Differences in pSTAT3 and C/EBPβ were confirmed by immunohistochemistry studies (Figure 8B) and Western blotting techniques (data not shown) and preliminary data are shown for HIF1α protein accumulation to support gene expression findings. Treatment of either CD33^+ ^or CD11b^+ ^tumor-cell line-induced MDSC with lipopolysaccharide, a known activator of MDSC function [39], caused further up-regulation of STAT3, C/EBPβ, and HIF1α concurrent with increased expression of ARG-1, iNOS, and NOX2-component NCF1 (data not shown). These results further support a role for these transcription factors in promoting human MDSC suppressive function. While suppressive abilities in both CD11b^+ ^and CD33^+ ^subsets correlated with increased expression of STAT3, C/EBPβ, and HIF1α, the dominant transcriptional pathway may be different. Indeed, therapeutic reversal of CD11b^+ ^or CD33^+ ^MDSC-mediated suppression corresponded with different transcriptional changes.

### Inhibitors of MDSC function show differential activity on MDSC subsets

As reviewed by Lechner and Epstein [40], tyrosine kinase inhibitor Sunitinib and all-*trans *retinoic acid (ATRA) have previously been shown to inhibit MDSC [26,33]. Studies in our laboratory have also identified celecoxib (CXB) and analogs dimethyl celecoxib (DMC) [41] and unmethylated celecoxib (UMC) [41] as inhibitors of suppressive function in CD33^+^, but not CD11b^+^, MDSC *in vitro *(Figure 8C). Of note, the reversal of MDSC effects by CXB and analogs DMX and UMC does not appear to rely upon cyclo-oxygenase (COX)2 enzyme inactivation, as demonstrated by the persistence of therapeutic effects in the presence of prostaglandin E_2 _rescue, efficacy of analog DMC with low to absent COX inhibitory action, and the absence of effect seen with the structurally-unrelated COX2-selective inhibitor naproxen (Figure 8C). Gene expression patterns in ATRA, Sunitinb, or CXB-treated CD33^+ ^or CD11b^+ ^human MDSC were used to understand better factors promoting suppressive function in these cells. As shown in Figure 8D, functional inhibition of human CD33^+ ^MDSC by ATRA, Sunitinib, and Celecoxib correlated with decreased STAT3 and HIF1α transcription. In comparison, functional inhibition of human CD11b^+ ^MDSC by ATRA and Sunitinib correlated with decreased C/EBPβ levels, but no change in STAT3 and HIF1α mRNA levels. Celecoxib was not found to have inhibitory actions on CD11b^+ ^MDSC and it was not observed to decrease C/EBPβ levels in this population. While preliminary, these data suggest that HIF1α, STAT3, and C/EBPβ may be key transcription factors related to suppressive function in tumor cell line-induced human MDSC, as was recently demonstrated for murine MDSC, and warrant further studies at the protein level as master regulators of suppressive activity with differential effects of human MDSC subsets.

## Discussion

Human MDSC comprise a diverse and complex group of suppressive cells that have been poorly characterized to date. Their accumulation and suppression of T cell responses in cancer patients, however, are quite clear and remain a barrier to successful cancer immunotherapy. In this study, using a new model for *in vitro *generation of tumor-associated human MDSC, we describe MDSC induction as a universal feature of human cancers and identify two distinct subsets of MDSC.

Studies to characterize human MDSC have been limited by the primary accumulation of these suppressor cells in individuals with significant illness (*i.e*. cancer, sepsis, trauma) and relative absence in healthy individuals [6]. In our laboratory, induction of human MDSC from healthy donor PBMC by a one-week co-culture with select human cancer cell lines has allowed the generation of highly pure populations of MDSC in significant quantities for characterization studies and functional evaluation with autologous donor T cells. Using this induction method, we evaluated over 100 human solid tumor cell lines for the ability to induce canonical CD33^+ ^human MDSC from healthy donor PBMC and found that these suppressor cells could be generated by tumor cell lines of all histiologic types, with the notable exception of breast carcinomas regardless of their HER2 and hormone receptor positivity. This finding prompted us to look for the induction of a different MDSC subset, and indeed we found that many tumor models with absent or poor CD33^+ ^MDSC induction preferentially generated CD11b^+ ^MDSC. Taken collectively, these data indicate that induction of MDSC is a common feature of human cancers and as such their presence may have a role in cancer detection and monitoring.

Using this model system, we then probed the pathways of induction and functional characteristics of these two cancer-associated MDSC subsets. Combining our previously published cytokine and gene expression data [16] with new gene expression, cytokine-induction, and antibody neutralization studies presented here, we identified IL-6, IL-1β and GM-CSF as the major inducing factors of CD33^+ ^MDSC and FLT3L and TGFβ as major contributors to CD11b^+ ^MDSC induction. Although generated by different tumor co-culture conditions, these two subsets appear to show at least partial overlap in morphology, phenotype, and function. Compared with their normal, non-suppressive myeloid counterparts, CD33^+ ^and CD11b^+ ^MDSC both showed immature myeloid morphology, low HLA-DR expression, and lacked lineage mature surface markers. MDSC have multiple mechanisms by which they can suppress T cell effector responses, and both CD33^+ ^and CD11b^+ ^subsets of MDSC showed up-regulation of canonical suppressive mechanisms (ARG-1, iNOS, NOX2). Previously, we demonstrated that subtle variations emerged in the patterns of suppressive genes that were up-regulated in human myeloid suppressor cells by different cytokine mixtures associated with active suppressive function [16]. Similarly, human MDSC induced by a range of human solid tumor cell lines exhibited small differences in the up-regulation of suppressive genes that likely result from subsets within the broadly defined myeloid suppressor cell population. Of note, some tumor models were found to induce both CD33^+ ^and CD11b^+ ^MDSC subsets, while others induced only one or neither population. Stratification into CD11b^+ ^and CD33^+ ^subsets showed greater arginase activity in the CD33^+ ^subset and partial overlap of function. These results likely reflect the complexity of myeloid suppressor cells, and will require finer dissection in future studies.

**Figure 9:**
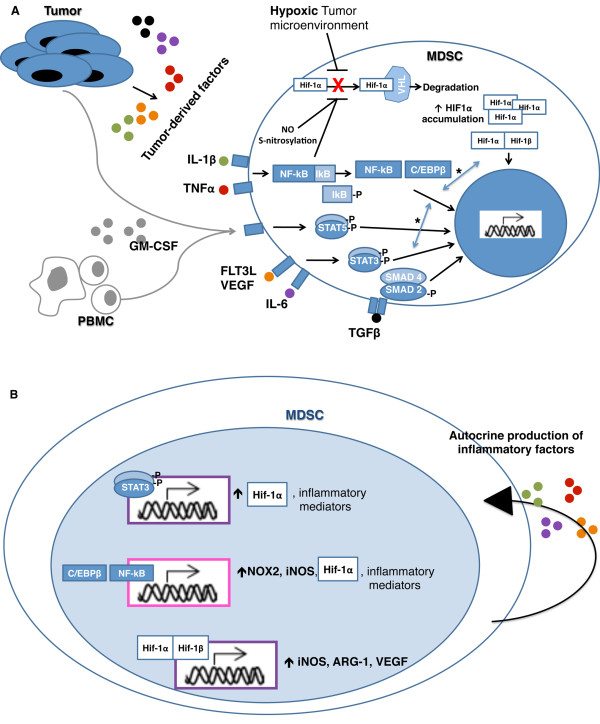
**Schematic for the induction of human CD33^+ ^and CD11b^+ ^MDSC in cancer**. *A*, Hypoxia and tumor-derived cytokines IL-1β, IL-6, TNFα, VEGF, FLT3L, and TGFβ in the tumor microenvironment promote signaling through STAT3, NFκB/C/EBPβ, SMAD2/4, and HIF1α pathways in myeloid cells. In addition to oxygen-dependent HIF1α regulation, inflammatory cytokines up-regulate HIF1α transcription (via PI3K or MAPK) and NO stabilizes HIF1α protein (via S-nitrosylation). Other factors influencing MDSC function include PBMC and tumor-derived GM-CSF, which supports expansion of myeloid progenitors and survival of MDSC, and IFNγ, which contributes to MDSC activation. Transactivation (*) between JAK/STAT, HIF1α, and NFκB signaling pathways amplifies the induction effects of tumor-derived cytokines and hypoxia in MDSC. *B*, Activated transcription factors translocate to the nucleus where they up-regulate expression of suppressive genes (iNOS, NOX2, ARG-1, VEGF) and autocrine production of putative MDSC inducers (*e.g*. IL-6, IL-1β, TNFα, and VEGF). The transcription factors driving suppressive function (and by extension potential therapeutic targets) in human MDSC appear to vary by subset, with a dominant role for STAT3 and HIF1α in CD33^+ ^MDSC (purple) and a dominant role for NFκB-C/EBPβ in CD11b^+ ^MDSC (pink).

The multiple pathways for induction and functional overlap of these MDSC subsets likely reflect a highly evolved, physiologic mechanism for tempering exuberant immune responses and preventing autoimmunity that is pathologically co-opted by some tumor cells to escape immune destruction. Indeed, inflammatory pathways appear to be major drivers of the suppressive functions in human MDSC induced by tumor cell lines and should be investigated as means of MDSC generation in sepsis and trauma patients where elevations of IL-6, IL-1β, and TNF-α are common and possibly are driven by the hypoxic environment of these conditions [15,42]. Given their pleotropic mechanisms of induction and suppressive actions, human MDSC will be difficult to inhibit for cancer therapy. A better therapeutic approach, then, is likely to evolve from inhibition of the transcription factors promoting the suppressive phenotype. Here we showed that HIF1α and STAT3 are critical transcription factors in CD33^+ ^human MDSC and C/EBPβ in CD11b^+ ^MDSC, respectively, and that effective inhibition of these subsets is accompanied by selective down-regulation of these transcription factors. These data suggest that therapies seeking to inhibit human MDSC at the level of conversion from normal myeloid cells will need to target multiple paths of induction occurring through STAT3, HIF1α, and/or C/EBPβ. These studies also highlight a potential means of high-throughput screening for MDSC-targeted therapies using the down-regulation of STAT3/HIF1α or C/EBPβ as correlates of inhibited suppressor function. Lastly these studies suggest that CD33^+^HLA-DR^low^HIF1α^+ ^and CD11b^+^HLA-DR^low^C/EBPβ^+ ^are highly specific phenotypes that may be used to isolate and study MDSC in cancer patients. From this investigation, we are able to propose a model for the induction and function of two key MDSC subsets generated in the cancer setting (Figure 9A and 9B). This model encompasses a role for inflammatory mediators, tumor-derived cytokines, and hypoxia in activating STAT3, SMAD2/4, NFκB, and HIF1 signaling in myeloid cells [15,26,38,43-46]. Signaling through and transactivation among these pathways yields up-regulation of key suppressive gene products related to MDSC function, as well as activation of autocrine or paracrine induction pathways to maintain and expand this population [15,44,45,47-50]. We highlight differential expression of STAT3/HIF1 α and C/EBPβ in the CD33^+ ^and CD11b^+ ^subsets, respectively, that may aid other investigators in therapeutic targeting, subset expansion, or MDSC monitoring in cancer patients.

## Conclusions

**Figure 10:**
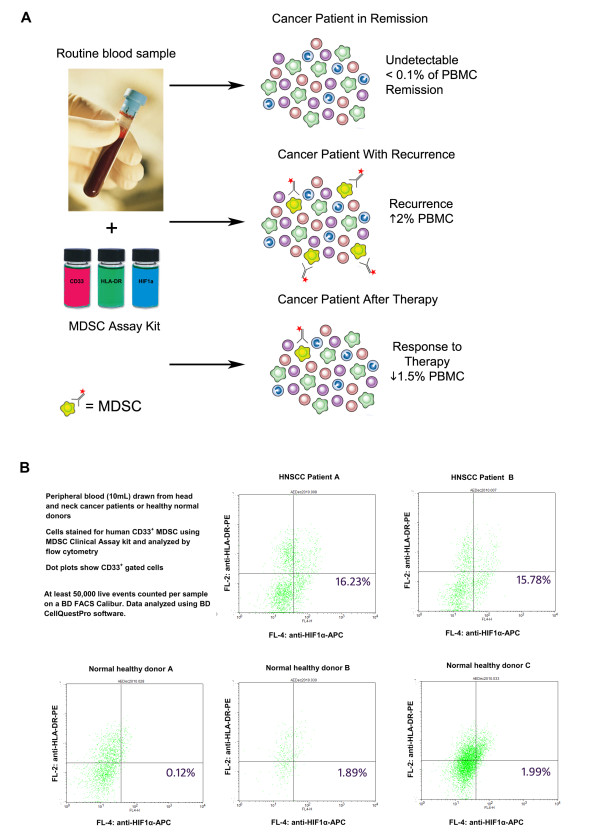
**Novel, minimally-invasive clinical assay for cancer detection and monitoring using MDSC biomarkers**. *A*, Schematic showing a novel, minimally-invasive clinical assay for cancer detection and monitoring. Patient peripheral blood cells are analyzed by routine flow cytometry for the presence of myeloid-derived suppressor cells (MDSC) as a marker for tumor presence. Active MDSC are distinguished from normal blood cells by a unique 3-marker phenotype that correlates directly with suppressive function. Accumulation of active MDSC correlates directly with disease stage and tumor burden, allowing physicians to track disease stage, tumor response to therapy, and tumor recurrence or progression by a simple blood test. *B*, Preliminary data demonstrating the CD33^+ ^MDSC subset in the peripheral blood of head and neck cancer patients using a recently identified phenotype: CD33^+^HLA-DR^low^HIF1α^+^. Ten milliliters of peripheral blood was collected from normal, healthy volunteers or HNSCC cancer patients under Institutional Review Board-approved studies HS-06-00579 and HS-09-00048. Cells were stained for CD33^+ ^and HLA-DR^+ ^using fluorescence-labeled monoclonal antibodies, then cells were fixed and permeabilized for intracellular staining of HIF1α by a third antibody. Stained sample PBMC and isotype controls were analyzed on a FACSCalibur flow cytometry using CellQuestPro software and collecting 50,000 live leukocyte events. CD33^+^HLA-DR^low^HIF1α^+ ^cells were found to be 15.78-16.23% of myeloid cells in cancer patients compared with 0.12-1.99% in healthy controls.

This study is significant for its broad analysis of human MDSC generation by a range of different cancer types represented by human tumor cell lines. MDSC generated by co-culture methods were then characterized for morphology, phenotype, gene expression and function. These data and methods provide an important pre-clinical tool for other investigators to examine other aspects of human MDSC biology and the development of MDSC-directed therapies. Furthermore, from these analyses two simplified phenotypes were identified that distinguish functionally suppressive human MDSC from normal myeloid cells. One potential use of these MDSC biomarkers is the detection of human MDSC in cancer patients as a means to track disease progression and response to therapy. Diaz-Montero and colleagues [30] initially suggested that human MDSC levels correlate with disease stage and preliminary data from an on-going clinical study in our laboratory suggests that MDSC detection in peripheral blood using definitive biomarkers for CD33^+ ^and CD11b^+ ^subsets can distinguish cancer patients from healthy individuals (Figure 10). In conclusion, we show MDSC induction to be a universal feature of human solid tumors and present a novel model system for pre-clinical studies of this important regulatory cell population.

## Abbreviations

(ARG-1): arginase-1; (C/EBP) β, 5- (and 6-): CCAAT/enhancer-binding protein; (CFSE): carboxyfluorescein diacetate succinimidyl ester; (c-kit L): c-kit ligand or stem cell factor; (COX2): cyclo-oxygenase 2; (FLT3L): fms-related tyrosine kinase 3 ligand; (GAPDH): glyceraldehyde 3-phosphate dehydrogenase; (GM-CSF): granulocyte-macrophage colony stimulating factor; (HIF-1α): hypoxia inducible factor-1 alpha; indoleamine 2,3-dioxygenase; (iNOS): inducible nitric oxide synthase;(IFNγ): interferon gamma; (IL): interleukin; (M-CSF): macrophage colony stimulating factor;  (MDSC): myeloid-derived suppressor cells; (NOX2): NADPH oxidase; (NFκB): nuclear factor kappa B; (PBMC): peripheral blood mononuclear cells; (PGE): prostaglandin E2; (Treg): regulatory T cells; (STAT3): Signal transducer and activator of transcription 3; (TGFβ): transforming growth factor beta; (TNFα): tumor necrosis factor alpha; (VEGF): vascular endothelial growth factor-a.

## Competing interests

A.L.E. is a co-founder and was previously part-owner of Cancer Therapeutics Laboratories, Inc. (Los Angeles, CA). All other authors declare that they have no conflicts of interest.

## Authors' contributions

MGL designed the study and wrote the paper. MGL and CM developed the methods, screened the tumor cell lines, and performed phenotype and gene expression studies. SMR, BB, TW, and NA assisted with these studies. ALE contributed to method development, supervised the studies, provided tumor cell line bank and maintained cell lines, and assisted with data interpretation. All authors reviewed the paper.

## Note

^1^Russell SM, Lechner MG, Gong L, Megiel C, Liebertz DJ, Masood R, Correa AJ, Han J, Puri JK, Sinha UK, Epstein AL. **USC-HN2, a new model cell line for recurrent oral cavity squamous cell carcinoma, with immunosuppressive characteristics**. *Oral Oncology*, in press.
